# ‘This place isn't worth the left boot of one of our boys’: Geopolitics, militarism and memoirs of the Afghanistan war

**DOI:** 10.1016/j.polgeo.2012.10.006

**Published:** 2012-11

**Authors:** Rachel Woodward, K. Neil Jenkings

**Affiliations:** School of Geography, Politics & Sociology, Newcastle University, Newcastle upon Tyne NE1 7RU, UK

**Keywords:** Afghanistan, Memoir, Military, Militarism, Popular geopolitics, *Testimonio*

## Abstract

This paper argues for the continued significance of the text as a source and focus in critical geopolitical inquiry. It establishes the utility of the military memoir in explorations of popular contemporary geopolitical imaginaries, and considers the memoir as a vector of militarism. The paper examines the memoirs written by military personnel about service in Afghanistan with the British armed forces, specifically about deployments to Helmand province between 2006 and 2012. The paper explores how Afghanistan is scripted through these texts, focussing on the explanations for deployment articulated by their authors, on the representations they contain and promote about other combatants and about civilian non-combatants, and the constitution and expression of danger in the spaces and places of military action which these texts construct and convey. The paper then turns to consider how a reading of the military memoir with reference to the genre of *testimonio* might extend and inform our understanding and use of these texts as a source for exploring popular geopolitics and militarism.

## Highlights

► Military memoirs are an under-utilized source in critical geopolitical analysis. ► Reading memoirs through the lens of *testimonio* develops understanding of the genre. ► Memoirists' rationales for deployment question the logic of the war. ► Portrayals of other personnel and civilians emphasise British military specificity.

This paper is about the continued salience of textual sources in critical geopolitical inquiry, and about the insights brought by one particular type of text – the military memoir – to the exploration of popular geopolitical imaginaries of war and to understanding militarism. We examine the published memoirs of British military personnel about their participation in the Afghanistan war, and offer a reading of and approach to these texts in terms of the understanding of the geopolitical context and the rationalisation of the use of military force which they collectively promote.

Military memoirs merit critical scholarly attention for two reasons. First, they are popular geopolitical texts used as source materials for civilian public understandings of war. They are distinctive because of their claims to veracity, and because the book format enables a quantity and degree of reflection about armed conflict and the experience of putting into practice the geopolitical objectives of the state from the perspective of an individual participant. They are distinctive too because they reflect a process of collaborative production in terms of writing, editing, marketing and producing a material object, the book, for public consumption; they can be viewed both as an individual's account but also as the outcome of collective enterprise between a serving or former member of an armed force and the publishing industry. Second, these books have a potential function as vectors of militarism. Militarism is the extension and prioritisation of military objectives and rationales into civilian cultural, social, political and economic life. Military memoirs facilitate this through their validation of the idea of the deployment of military power. They may facilitate war, not in any crude causal manner, but as artefacts of cultural militarism through which military interventions become normalised, simplified and potentially sanitised, and justified. The potential militarising effects of these memoirs is not, however, a simple, linear or absolute process, and whilst collectively they can be understood as vectors of militarism, if more closely considered we see the disruptions they bring to dominant discourses which prioritise military solutions and interventions.

Since 2001, initially in response to the 9/11 attacks, military personnel from many national armed forces have been deployed to Afghanistan, operating under UN and subsequently NATO command or mandate as the International Security Assistance Force (ISAF) on a securitization and stabilization mission. The ISAF deployments were justified by international bodies and national governments as necessary for the elimination of radical Islamist political and military control in the country because of the effect this was deemed to be having on regional and global instability and insecurity, not least because of the significance of a Taliban-controlled Afghanistan in the support of international terrorist networks. Initially confined to Kabul, under NATO command from 2003 ISAF's role was extended to cover the whole of Afghanistan by mid-2006, and this involved both attempts at provincial reconstruction and the deployment of armed forces. The ISAF deployments were violently resisted by those opposed to the establishment by the US-supported government of Mohammed Karzai, and the presence of foreign troops. The resistance by groups commonly identified by NATO as Taliban (in practice, a label used to identify a range of opposition groups and forces which may or may not share a unified, common set of objectives beyond opposition to foreign intervention) has been met by complex counter-insurgency operations, and is on-going as we write. Troops from fifty contributing countries have rotated in and out of Afghanistan for ten years; in 2012, the US contingent stood at 90,000, UK troops numbered 9500 and Italian and French contingents comprised around 3000 personnel each. Additional contributing nations also included Canada, Australia, the Netherlands, Germany and Denmark, and participating military forces included those involved in both direct combat and in non-combat roles, and in mentoring and operating with the newly established Afghan National Army (ANA) and Afghan National Police (ANP) forces. The violence in Afghanistan has been extremely costly in lives and livelihoods for all those directly and indirectly involved. The UN Assistance Mission Afghanistan (UNAMA) reported 3021 civilian fatalities as a consequence of military operations in 2011, 2790 in 2010 and 2412 in 2009, with the caveat that civilian fatalities are almost certainly under-reported (UNAMA, 2012). Civilian casualty rates are higher, and an unknown number of Afghan civilians have been affected through forced migration away from conflict zones, the consequences of political instability, and negative effects of the disruption to economic activities, particularly food production and distribution, and to healthcare. As of June 2012, British military fatalities stood at 415 since October 2001, with around 2000 serious casualties wounded in action (MoD, 2012). Total ISAF coalition fatalities number over 3000.

As with other conflicts this war has produced its own literature, and between 2007 and 2012, 24 military memoirs have been published by 21 serving and former members of the British armed forces about their experiences in Afghanistan. These books had an estimated market value of just under £5 million by spring 2012 and between them have sold over 500,000 copies (figures from Nielsen Bookscan, 2011). This paper is about these books. We do not include here the accounts written by journalists and other commentators covering the war and drawing on first-hand experience as non-combatants. Neither do we include here accounts by military personnel serving with other national forces. Our focus is explicitly on British military memoirs of the Afghanistan war.

In this paper, we start by outlining the role of the military memoir in the study of war, in the popular geopolitical imaginary and in the critical geopolitical project. We then explore three specific aspects of the geopolitical imaginary articulated and reproduced within the military memoirs of the Afghanistan war, looking at their explanations of the rationale for deployment; their portrayals of the combatants and non-combatants against and with whom their authors are deployed; and the representations of the places and spaces of conflict through which the geopolitical imaginaries they communicate are constituted. We conclude with an argument for the continued salience of the text within critical geopolitical inquiry, drawing on the ideas suggested by the genre of *testimonio* to indicate how a more nuanced understanding of the military memoir, alert to its communicative intentionality and collectivity in origin and production, might extend our understanding of that key geopolitical actor, the soldier, and of the processes through which militarism operates, takes effect and is potentially also countered.

## Military memoirs and popular geopolitics

Military memoirs are the first-person accounts of experience in armed conflict produced by military personnel. The centrality of personal revelation about war is an organising principle for the genre (Harari, 2008). This revelatory core, which may be idealistic and supportive of intervention or more condemnatory about a war's futility, receives its validity and authority through lived experience, what Harari calls the ‘flesh-witnessing’ of the author. This embodied experience provides the military memoir with both its privileged status as a factual record of a specific war or incident within that, and its more contingent status as a personal and thus partial and uniquely positioned account.

War brings an equality of opportunity for suffering, and there is some debate whether, socially and politically, the account of the direct combatant is or should be privileged in the study of war (Hynes, 1997), or whether the range of experiences of all those caught up in armed conflict should be included (Vernon, 2005). That we focus here exclusively on the accounts of a small number of British military personnel in an asymmetric conflict affecting so many, should not be taken as dismissive of those other stories (and we recognise their absence here). Our focus, however, is on soldiers' published stories, because of the specificity of combatants' perspectives and their role in shaping and maintaining specific, British popular understandings of the Afghanistan war and the utility of military force.

Our conceptual approach to these memoirs emphasises their role, individually and collectively, in the cultural delivery and consumption of understandings of war (McLoughlin, 2011). Our analytic approach prioritises the functionality of the autobiographical text, as something to be studied primarily for what it can do, as much as for the intricacies and logics of its linguistic and structural form (Smith & Watson, 2010). We are interested in how autobiographical narratives ‘encode or reinforce particular values in ways that may shape culture and history’ (Couser, 2005: 129–130), and how these military memoirs might work as a source for critical geopolitical inquiry.

The origins of this paper lie in a research project on the production of military memoirs. The wider project explored the contemporary British military memoirs published since 1980 with a view to establishing how these books came to be written and published (see Woodward & Jenkings, 2011a). Over 150 memoirs published by people who had served with the British armed forces were collected and read, their form, paratext and wider public reception in terms of sales and reviews analysed, and a sub-set of authors interviewed to explore the motivations, rationales and constraints on producing and publishing a memoir. A small number of publishers were also interviewed. This paper draws on that project and includes data from the 24 Afghanistan memoirs published to July 2012, and semi-structured research interviews with six authors of Afghanistan memoirs, selected to provide accounts across the range of authorial experiences of memoir writing. The project did not set out to provide a definitive international comparison in military memoir traditions and practices between national forces (see Kleinreesink, 2012), but we have observed how the British memoirs about Afghanistan are framed by an awareness (plainly evident in British media responses to the war) of public sensibilities sympathetic to the idea of the affective and physiological costs of war on the minds and bodies of soldiers, and celebratory of the military operative as heroic (Woodward, Winter, & Jenkings, 2009). This mediated public discourse is also inflected with criticism of the political management of the war and the armed forces, yet often falls short of outright rejection of the idea of war in its performance of support for the military personnel commanded to execute the violence demanded by the state in pursuit of its wider geopolitical objectives, and the British Afghanistan memoirs reflect that. See, in contrast, the US memoirs of Afghanistan which are more celebratory of the military and military exceptionalism, and which have developed in tension with the literature and memoirs of the Vietnam War (Kieran, 2012).

Neither did our project set out to determine over a longer timescale the precise differences between the more recent memoirs from Afghanistan and indeed Iraq, and those from earlier wars involving British personnel. However, when historicizing the contemporary memoir it is evident that the personalized revelatory trope so defining of the genre (see Harari, 2008), whilst still present, is increasingly being supplemented and surpassed by a more collective mode of representation directed at the possibility of soliciting a shared ethical and political response to war amongst the readers of memoirs (and we return to this issue below when we consider the Afghanistan memoirs with reference to *testimonio* literature). More immediately obvious is the fact that all the Afghanistan memoirs in our collection have been written and published whilst the war is still on-going. In contrast to the memoirs from other wars of the twentieth century involving British personnel, which include both world-wide conflict (the First and Second World Wars and the Cold War) and smaller campaigns such as Suez, Oman, Northern Ireland, the Falklands, the Persian Gulf, Bosnia and Sierra Leone, the Afghanistan authors have no knowledge of how the war will end. The Afghanistan narratives therefore inevitably avoid closure around the question all memoirs pose implicitly, about the wider costs and benefits of military violence and of the broader strategic picture, and focus instead on smaller, personal incidents where the tactical activities of small groups are brought to the fore.

As published texts benefitting from public commercial distribution, military memoirs have always shaped public understandings of war (see Fussell, 1975; Lunn, 2005; Winter, 2006), and the mechanisms and extent of influence remain an open question (Ashplant, Dawson, & Roper, 2000). For military memoirs, pertinent issues include perceptions of the authority and platform of the writer (Thompson, 2010), their rank, status, personal history and experience, and the contingency of reader responses according to his or her time and place of reading (see Hones, 2008). In this paper, we acknowledge the potential for diverse readings of the texts we discuss, whilst (necessarily) prioritising interpretations of these texts informed by our own readings and wide experience of the genre, by authors' understandings and experiences of engaging with their readers, and also by public commentaries and reviews of these texts as they have appeared on the market. Most significantly for the argument which follows, we are concerned with the work of the texts and the authors in encouraging a particular kind of response to the text, underpinned by an observation that for all the diversity within the sub-set of memoirs dealt with here, there remain certain salient and common ideas providing the genre and the sub-set with its unity of intent to communicate a personalised experience to a readership understood by authors to be lacking in knowledge about that experience.

Military memoirs have also shaped scholarly understandings of war and military participation. Within sociological analyses of the military, memoirs have been used as sources of empirical information for understanding the forms, functions and spatialities of military masculinities (Duncanson, 2009, 2011; Higate, 2003; Woodward, 1998; Woodward & Jenkings, in press-a), the practices of military communication and cohesion strategies (Baker, 2010a, 2010b; King, 2006; Kinzer Stewart, 1991), the organisation of military capabilities (King, 2009, 2010), the articulation of citizenship ideas amongst military personnel (Woodward, 2008) and the understanding of mental and physical trauma and narratives of trauma in war (see Robinson, 2011, 2012; Woodward & Jenkings, in press-b). As cultural documents, they invite analysis for the ideas they promote about distinct groups such as Elite and Special Forces (Connelly & Willcox, 2005; Newsinger, 1997), and inform popular ideas about military life so influential in recruitment (Gee, 2007).

Military memoirs are also geopolitical texts and thus have on occasion constituted a useful source of tactical and strategic insight for the exploration of geopolitical narratives around specific conflicts. The Afghan wars fought by British imperial forces in the 19th and early 20th century, for example, draw on a range of military participants' memoirs to inform Fremont-Barnes' (2009) analysis of those conflicts. Newsinger's (1994) analysis of British army memoirs from the Malayan conflict shows their role in the construction of a wider, celebratory discourse of British military prowess and imperial superiority. Analyses of representations of the Falkland Islands in the aftermath of the Falklands/Malvinas conflict (Arquilla & Rasmussen, 2001; contributions to Aulich, 1992; Dodds, 1996, 1998; Foster, 1997, 1999) draw on the detail provided by memoirs to complement broader arguments about the construction of geopolitical discourses circulating around that war, as do analyses of Bosnia and the Bosnian war (Baker, 2011; Robison, 2004; Simms, 2001).

But memoirs have been under-utilised as a source within the study of popular geopolitics (and a similar observation has been made about their absence in anthropological studies – see Brown & Lutz, 2007). Many within the genre have high public visibility through large sales, and go on to inform much broader civilian discourses about conflicts which circulate in the public domain (the books and personae of Andy McNab or Chris Ryan are examples). Yet they have not had the equivalent purchase on the study of popular geopolitics as other cultural forms such as film and gaming. We can speculate on the reasons for this. It may be that the experiences recounted are uncongenial (Newsinger, 1994). There is also a wider issue about the relative lack of engagement with the figure of the warrior and his or her activities within contemporary geographical scholarship (Dalby, 2008). We should be alert to the limits as well as the possibilities of life-writing, which influences memoirs' utility to scholars (Smith & Watson, 2010). Also, although often entertaining, military memoirs are not marketed as entertainment and though they sell, they don't have the market visibility or profile of other popular cultural forms within the military-entertainment complex.

As a source for the study of popular geopolitical understanding, military memoirs sit beside other media, literary and entertainment forms in that they are essentially representational. In recent times, the textually-orientated, representation-focused approach of critical geopolitics has been challenged by arguments for a renewal of approaches to popular geopolitics more attentive to its lived, experiential dimensions and less pre-occupied with the text as a central focus. Suggestions include the incorporation of emergent conceptual approaches of feminist geopolitics, non-representational theory and consumption studies with their foregrounding of the daily practices of people's lives (see Dittmer & Dodds, 2008; Dittmer & Gray, 2010), for methodological and ethnographic approaches which pay greater attention to people's experiences and every day understandings (Megoran, 2006), and a closer consideration of the practice as well as language-based effects of geopolitical discourse (Müller, 2008). But like Dalby (2010), we are reluctant to part with our texts and their authors just yet, not least because of their centrality to popular military and geopolitical imaginaries in general, and in the case of the Afghanistan war, their key position in articulating ideas about the morality and meaning of that particular war in the absence of wide media coverage and the limitations of blogs. Like Dalby, we see the unpacking of militarist mappings of global space as the core concern of the critical geopolitics project, and we see the military memoirs as key (and hitherto under-explored) sources for understanding how rationalisations of military power and the legitimization of military action are articulated.

There is a further point here about the continued salience of the text, and the significance of the memoir. If it is the case that popular geopolitics, as a set of practices within the social world, provides legitimation for, and acquiescence to, the politics of militarism which in turn are the outcome of elite scriptings of the world (Dittmer & Gray, 2010), then texts would seem to us to remain a crucial area for critical study. Within the genre (and within the Afghanistan memoirs as a sub-set), there is considerable diversity in the backgrounds (social, military) of the authors. Some are recognisably elite and indeed personally responsible for the ‘scriptings of the world’ that Dittmer and Gray identify. We concur with Dalby (2010) that if we are interested in war and conflict, we need to be interested in those charged with the conduct of war from positions of power. Whilst not necessarily the ‘intellectuals of statecraft’ responsible for formal state geopolitical discourse, some military authors are, by virtue of their senior position in the chain of command, still responsible for the construction of geopolitical imaginaries and geopolitical strategies at the behest of the state (General Dannatt (2010) and Colonel Tootal (2009) are examples). Furthermore, some of the most vehement critique about the moral irresponsibility and practical inadequacy of military intervention in Afghanistan comes from those very elites – colonels and captains – whose scriptings of the world have been seen as problematic for their dominance in the popular geopolitical imaginary.

But some memoirists, quite simply, are not ‘elite’. The criticism that the study of popular geopolitical discourse is ‘somewhat inexplicably still focused on the elite visions of media moguls, movie directors, and lower-level yet still relatively empowered media functionaries like writers and reporters’ (Dittmer & Gray, 2010: 1664) becomes more problematic when that vision originates, as many of these memoirs do, in the post-operational writings of a non-commissioned soldier or junior subaltern seeking resolution in the aftermath of war. These books all start with a real person. These people provide personal, experientially-based accounts of engagement with armed violence, and reflections on the legitimation of and acquiescence (or otherwise) to the politics of militarism and the wider geopolitical ambitions of the state, from a position of relative subordination.

Within our collection of 24 books and 21 authors there is considerable diversity (and Table 1 provides an overview of the collection). The books we consider mostly revolve around a central authorial experience of deployment with ISAF to Helmand province from April 2006 onwards on the various rotations of Operation Herrick (although some also interweave other stories from other theatres of war). In terms of the military careers and origins of the authors, six were deployed by the Royal Navy – one (Orchard) through the Fleet Air Arm and five (Croucher, Farthing, Hammond, Ormrod and Olafson) as Royal Marines. One (Duncan) was deployed with the Royal Air Force. The remaining fourteen deployed with the British Army – two with the Army Air Corps (Macy and Madison), one as a Combat Medical Technician (Taylor), one as a Joint Terminal Attack Controller (JTAC) (Grahame), nine (Beattie, Bury, Cartwright, Compton, Docherty, Flynn, Hennessey, Scott and Tootal) deployed in infantry roles, and one (Dannatt), as Chief of the General Staff, was not deployed on operations. In rank, the authors range from General to private soldier or equivalent, with the majority being senior Non-Commissioned Officers or junior officers. Two of the authors (Madison and Taylor) are women, and one (Olafson) is a Canadian national serving with the British armed forces. Twenty of the twenty-four memoirs could be classified unproblematically as narratives of operations and fit clearly with genre of the military memoir as we have defined it. Two memoirs cross over into the genre of the trauma/recovery narrative (both Ormrod and Compton write primarily about their healing journeys from traumatic injury), one is primarily a career autobiography (Dannatt) and one is marketed and identified by its author (Farthing) as a ‘dog book’, because *One Dog at a Time* documents a Royal Marine's attempts to rescue stray dogs in Helmand whilst serving there on operations. Bestsellers include Macy (over 120,000 copies sold of both books), Hennessey (around 75,000 copies), Beattie (c.70,000), Farthing (c.50,000) and Tootal and Croucher (c.20,000 each) (figures extrapolated from Nielsen Bookscan, 2011). A number of the books intersect with other media formats such as tabloid newspapers, television documentaries, and (in terms of their marketing and design) with first-person shooter gaming. In terms of absences, we have yet to find memoirs in English detailing the Ghurkha Brigade experience with the British armed forces, despite their presence in Afghanistan. The prohibition on the publication of special forces memoirs in the mid-1990s continues, so despite high levels of activity the experiences of members of the Special Air Service and the Special Boat Squadron remain unpublished. The accounts of those in rear-echelon units and in logistics, communications, engineering, mechanical and intelligence units remain relatively unknown. We are dealing with a sub-set (primarily infantry and front-line roles) of a sub-set (English-speaking British forces in ISAF) in a specific area (Helmand within Afghanistan where up to 80,000 British personnel have deployed over successive rotations). The geopolitical imaginaries and explanations of the utility of military force, which provide our focus here, are not the sole messages which emanate from the ISAF experience in Afghanistan, nor even the totality of the British forces experience. Following our coding and analysis of the texts, we have selected for discussion here those themes which speak to the wider critical geopolitical project and have limited our discussion to those which figure most explicitly and frequently across the collection, these being the logic and utility of deployment, the nature of the combatants and non-combatants involved in the war, and the representations the memoirs construct of the place of deployment. It is to these that we now turn.

## Memoirs and the scripting of Afghanistan

The geopolitical imaginaries articulated and communicated through the texts of military memoirs speak to three core ideas. These are: the rationale for the deployment of armed forces; the portrayals of combatants and non-combatants against, with and amongst whom memoirists fight; and soldiers' perceptions of the spaces in which they fight as constitutive of the logics for the use of military force.

### Why are we here?

The diversity of texts recounting soldiers' tales in Afghanistan has already been indicated, and this includes variation in the extent to which the logic for deployment is articulated. At its simplest, this is a question of quantity of description: Beattie's account of a 13-day deployment in Garmsir fighting closely with ANA and ANP personnel returns repeatedly to the question of the rationale for deployment, whereas Grahame's account of his experiences as a JTAC is based on the briefest of explanations as to why he is there in the first place (Beattie, 2008; Grahame, 2010). These differences reflect the different logics of the narratives. Grahame's is a fast-paced series of recollections of action-in-the-moment from a range of contacts and events over the course of his deployment. Beattie's is a more reflexive exploration of the action and aftermath of a specific operation, and his post-deployment attempts to reconcile himself to his experiences of close combat are parallelled in the text with his attempts to reconcile the intention of securitization with the reality of insecurity in Garmsir at the time.

There is also a more complex issue here beyond the internal purpose within the text of the rationale for deployment, which is the communication of ideas to the reader about the rationale as a function of the text itself. Although deployed on Operation Herrick in small numbers from December 2001, the reorganisation of ISAF forces in 2005 led to an increase to around 6000 of British personnel in Afghanistan from April 2006 and their deployment to Helmand province in the south with the objective of its securitisation. Memoirs recording experiences of those deployed on Operation Herrick 4 (2006) frame the logic for deployment around official (i.e. British government) rationales of securitization for the purposes of stabilization and reconstruction of civilian political and physical infrastructure, and Tootal (2009), commanding officer of 3PARA, details both this and his initial belief in the plan. Docherty (2007), a Captain in the Scots Guards, notes his enthusiasm for the mission, his attempts to learn Pashtu, and his efforts through his operational mentoring and liaison work with the Afghan National Army (ANA) to understand the specifics and complexities of the reconstruction programme. He is ‘mustard keen’ (p. 48) on the central mission: ‘“nation-building” sounds fascinating and very honourable’ (p. 45). Scott, a 3PARA corporal who took part in many of the earliest military actions in Helmand, is representative of other lower-ranking personnel providing scant detail; we are told that the Brigade deployed ‘to make a difference to the ordinary Afghan’ (2008: 27) and his book starts by drawing on an end-of-tour speech by Brigadier Ed Butler, commander of UK forces in Helmand, celebrating the activities of the regiment, but there is a sense of absence around the rationale in this memoir beyond a vague idea of ‘hearts and minds’ as something to be captured amongst the civilian population.

Throughout the next two years, the experience of British forces in Helmand under successive troop rotations was one of high intensity, kinetic, counter-insurgency operations. Arriving at Camp Bastion, the British HQ in Helmand in spring 2008, Bury, a Second Lieutenant commanding a platoon of Irish Rangers, describes attending a briefing.

We are there for three hours. The situation is so complicated, there are so many tribal, cultural, political, religious and military dynamics, that I am overwhelmed. *How can we cope with this?* It seems that we soldiers, primarily trained to fight conventional wars, need to be friendly police, social workers, government representatives, aid workers, bomb detectors, engineers, killers, medics . . . the list is as endless as the problems we face. (Bury, 2010: 82)

The theory underpinning the counter-insurgency strategy of British forces is then explained:

A red map, all Taliban held, is projected of the Helmand River valley. Green dots show areas of ISAF control. This is the famous ink-blot strategy, a series of FOBs [Forward Operating Bases] out of which the West's calm, democratic and peaceful virtues are meant to flow like an expanding green ink sea, searching out other blots, joining up and turning Helmand into one happy, green place. We can see the basis of the plan on the map, but the green blots are only dots in a sea of red. (Bury, 2010: 83)

As his platoon adjust to the reality of the deployment, he knows that he has to squash the fears of men under his command (‘shove them out from the juggernaut's headlights that they seem caught in’ p. 84) and provide a rationale. He gives them a pep talk providing the official explanation about the necessity of defending the district centre in Sangin and providing security so that the government of Afghanistan can create conditions for further stabilization and consolidation.

We've heard it a hundred times. That statement is why we are here. We get the gist of what it means. Keep the Taliban out, show the people that the government can govern inside the blot, let the Afghans run things. Simple.

But it didn't really explain why we were here in the first place. (Bury, 2010: 85)

So he provides an alternative explanation to ‘the nonsense we've heard about why we're here and about the schools and women's rights’, a rationale hinging on how the presence of ISAF forces prevents Afghanistan being used as a base to attack home, and how stopping ‘some of the heroin getting back to Liverpool, Belfast, Dublin, then maybe less people will have their lives and the family's lives screwed up’ (p. 85). The rationale for deployment as a drugs control strategy clearly made sense to Bury as one of the very few believable explanations that he felt he could provide to men under his command. Similarly, Flynn sets a scene for the reader by introducing Helmand as ‘pretty much a giant drugs factory’ where the Taliban used a proportion of the income from the trade to fund military operations (2011: 15–16). Further rationale for the deployment is absent, and it is a feature of the Afghanistan memoirs how few provide detailed explanation of official government rationale for armed intervention in Helmand. Yet the narratives demand an underpinning logic (why else would there be war?), so for Flynn, this comes down to a simple fact that ‘fighting was a way of life for most of Helmand's male population’ who ‘shared a single aim: the burning desire to kill any foreign soldiers on their home turf’ (p. 9).

The absence in many of the memoirs of a clear political rationale for British forces' engagement is a function of a number of factors. Some authors are writing from the perspective of military operatives rather than strategists, and the absence of strategic planning within their military roles is reflected in their accounts (see for example Cartwright, 2011; Croucher, 2009; Farthing, 2010; Ormrod, 2009). As Scott puts it, ‘In 2006 we Paras were sent there to do a job, we don't ask the reasons why, we just get on with it’ (2008: 27). In other cases, this absence is a reflection of the sense expressed by Bury, of the lack of *meaningful* explanation in official discourse, and the sense therefore of having to find an alternative rationale. Beattie, in the conclusion to his memoir, muses on the moral necessity of those back home knowing what soldiers were doing and what they were suffering (2008). But he notes also the difficulties of this, that to fully understand the war, the British people needed to be presented with the context of the war. This in turn raises a central question for him: ‘…is it a country worth saving? In fact is it a country at all?’ (p. 297). These Afghan memoirs, then, provide an anti-geopolitical reasoning, locating the logic of individuals' actions away from the material and representational rationales provided by the state (although we would not claim these texts as a form of ‘anti-geopolitics’ – see Routledge, 2003).

We should note, though, that more forceful than the absence of strategic geopolitical commentary across this body of texts is a clear and unmistakable condemnation of the formal state geopolitics that resulted in the deployment of troops in the first place (Woodward & Jenkings, 2011b). Expressions of disdain are calibrated by rank. For Bury, a young subaltern, ‘it feels like we are a hammer being used to tighten a screw’ (2010: 107), ‘all we're doing here is helping these corrupt bastards with their power plays’ (p. 132), where in effect his men ‘are risking their lives every day. For this place? This place isn't worth the left boot of one of our boys’ (p. 129). For Docherty, a Captain who resigned his commission after his deployment and who received official censure for the publication of critical negative comments about the campaign,

…we've lost this campaign before it's even started. I know that soldiers dying in Helmand is pointless. I know that our artillery shells and Apache helicopters will, in a tragic replay of Soviet clumsiness, kill countless Afghan civilians while pursuing a nebulous enemy. I feel foolish for believing so wholeheartedly that in coming to Afghanistan I would be part of something intelligent, meaningful and constructive. I know now that our entry into Helmand has been ignorant, clumsy and destructive – a vainglorious folly. (Docherty, 2007: 178)

Tootal, a more senior officer who also resigned after his tour of Afghanistan, is clear in his critique that failures in strategic planning, in the problematic intersection between securitization and reconstruction, and in meeting political objectives locally at odds with national and international strategy, explain the fact that ‘We did not win the war, we did not bring about peace or reconstruction’ (2009: 255).

We should also note how within one specific memoir, a rationale for deployment comes to be created. Deployed with the Royal Marines to Now Zad, in the winter of 2008, Pen Farthing's (2010) memoir describes in and amongst the details of military activities the process of rescue and adoption of a number of dogs, individuals from a larger pack of strays which roamed the town or were used for fighting for sport. His memoir is about animal rescue, an activity which develops out of his military role and justified on the grounds that although, in his position, he could not contribute to reconstruction and development work directly, by engaging in animal welfare work including neutering programmes and education for vets, at least he could do something to help the people of the province, particularly children, at risk from rabies and dog bites from the multitude of strays in the locality. A memoir which appears at face value to be a whimsical dog story (‘*Marley and Me* with guns’, in the author's words), turns out to be the sole book in the collection with a positive outcome, where a meaning for the military presence is rescued like Farthing's stray dogs from a wider mass of contradictions inherent in the geopolitical reasoning that sent troops to Afghanistan in the first place.

### Who is here?

These memoirs are about people. If geopolitics is ‘a discourse or practice engaging in the creation of geographical relationships and orders so that global space becomes divided into simplistic categories such as good/evil, threatening/safe and civilised/barbaric’ (Dittmer & Dodds, 2008: 41), then military memoirs are surely textual strategies through which these binaries and categories are constituted and reproduced, and this is a social process undertaken with reference to people.

The demarcation of simplistic categories, which is a feature of popular geopolitics, is a necessary feature of the Afghanistan memoirs which starts with the identification of the enemy and the inscription onto this figure of specific attributes and characteristics. Needless to say, given the location of this war, an orientalist construction of an enemy Other is common. A number of memoirists (Croucher, Flynn, and Orchard are all examples here) set the scene for the reader by retelling stories of pre-deployment in-theatre briefings about enemy personnel, and these are necessary both for the narrative arc of the story (the reader needs to be told a bit about an enemy in order to legitimate the stories of the destruction of the enemy which follow), but also for the representational purposes of these stories of asymmetric warfare (the reader needs to be told that this is a specific enemy with distinctive characteristics which in turn merit particular armed responses). In these scene-setters, the idea is articulated of a dangerous enemy, bound by different (or absent) moral codes and rules of engagement, and possessed of (simultaneously) known yet unknowable capabilities and a lethal capacity for violence. Constructions of the enemy Other conform to the ideas predicted by the concept: he (always he) is strange, foreign, different, bestial, unjustified and vicious. Macy, an Apache pilot, introduces his reader to the Taliban as essentially and inhumanly vicious, describing in an opening chapter the mutilated bodies of Special Forces soldiers he sees on his cockpit video screen on a particular operation (2008). Indeed, for Flynn, this enemy is sufficiently Other to merit the use of a different type of weapon. As he puts it, whereas small calibre rifles such as the SA80 were fit for purpose against Soviet forces during the Cold War, ‘in Afghanistan, against enemy fighters who had to be stopped dead in their tracks first time every time, larger calibre weapons like the .50 cal were a much better idea’ (2011: 34). Flynn and Bury both provide portraits of an enemy rendered super-human and immune to pain by drug use.

Yet this is not an enemy to be dismissed because of its deviation from a Western military norm, and whilst this enemy is Othered, he is also necessarily respected. Croucher (2009) reports being told to under-estimate the Taliban at his peril, and Bury, in an accolade from one warrior to another, notes after one particularly long and difficult contact, that ‘after all we've thrown at them, their courage is admirable’ (2010: 231). This is an enemy which, the British soldiers recognise, learn from their observations and experiences, changing strategies and developing tactics as the war progresses in order to shape the conflict on their own terms. Just as British soldiers are trained to do.

This enemy is also invisible and unidentifiable, mysterious in ways that a state military force so clearly is not. They are rarely seen; the presence of Taliban fighters is indicated by small arms fire and rocket attacks, by muzzle flashes and tracer rounds, by obscured movements in distant tree-lines, by the IEDs they lay and by trails of blood left by the dead and wounded. Although occasionally seen through the sights of a rifle – Beattie (2008) describes in detail shooting an enemy fighter, his first direct kill in a long military career – more often they are not. To those watching from above in helicopters such as the Apache, with the power of aerial overview magnified by the technologies of military observation, enemy fighters are more visible but remain anonymous figures on a screen.

The enemy's unidentifiability extends to his unknowability. Memoirists report repeatedly on the fluidity of the category of ‘Taliban enemy’, which is presented as ranging from hard-core Islamist fundamentalists motivated by ideas of holy war, through to ‘$10 Talibs’, local farmers who have taken up arms in return for payment as and when required, prompted or coerced, or merely for self-protection. The continuum between fighter and civilian is further complicated by practices (again represented as indicative of an alien Other) of shifting allegiance compounded by local political expediencies whereby alliance or support may be granted by Afghan nationals to people ISAF forces identify as enemy. This fluidity is compounded by the cosmopolitan character of the Taliban alluded to by a number of writers. Cartwright (2011) talks of Chechen Taliban fighters, Flynn (2010) talks of ‘Brits, Saudis, Kuwaitis, Yemenis, Chechnyans, Pakistanis and your bog-standard home-grown Afghan variety’ (p. 260) and Bury (2010) and Taylor (2011) report rumours of Taliban fighters with Birmingham accents and Aston Villa tattoos (Aston Villa being a British West Midlands football club). There is also the problem of positive identification and not knowing what to look for. Beattie tells of his observation of various local people calling on the provincial governor of Helmand Province at his headquarters in Lashkar Gah. Many wear a distinctive black turban which he associates with the Taliban, but his interpreter repeatedly corrects him as they watch the stream of visitors, remarking that ‘Everyone knows who they are. And anyway they tie their turbans in a distinctive fashion’ (2008: 77). This confirms for Beattie and subsequently for the reader something of the complexities and unreadability of the political situation in Afghanistan. Later, during the attack on Garmsir, he views the body of a dead Taliban fighter noting the similarities in looks, clothing and equipment between the dead man, local civilian farmers, and ‘the policemen who were on my side’.

‘Punjabi’, said Gulzar [an ANP Colonel] from behind me.

‘How do you know?’

‘Because despite what you Westerners think we do not all look the same.’ (Beattie, 2008: 108)

A second prominent group of other people, who sit along the boundary between the familiar self and the threatening other, are the Afghan National Army (ANA) and Afghan National Police (ANP). These young institutions established by the Karzai government for securitization of the fledgling government, were funded, equipped, trained and deployed by ISAF forces, and a number of the Afghanistan war memoirs are essentially accounts of actions undertaken as part of the Operational Mentoring and Liaison Teams (OMLT), or in conjunction with OMLT-mentored units. The range of experiences of ANA and ANP contact reflects the diverse origins and motivations of members of these forces. Beattie, working alongside an ANP unit, comes to trust and respect one of its commanding officers, Major Shahrukh, and mourns his death in defence of an ANP position (Beattie, 2008; see also Duncanson, 2011; Woodward & Jenkings, 2011b). Farthing (2010), the dog-rescuing Royal Marine, succeeds in this precisely because of the intervention of an ANP commander, Rossi. But memoirs abound with tales of friction between the ANA and ANP, and between Afghan and British forces (Beattie, 2008; Bury, 2010), of their lack of operating procedures and systems (Hennessey, 2009), absenteeism amongst troops (Docherty, 2007), and cultural practices seen as strange and unmilitary, with suggestions of paedophilia and child abuse. Their sexuality is also questioned. Flynn reports a colleague's description that

They're rubbish: most of them spent the day lying around, smoking weed and planning who they're going to shag on Thursday night. As in, each other. Thursday night for the ANP is man-love night: we're talking eye-liner, flowers in the hair, jewellery, the lot.’ (Flynn, 2011: 27).

For all this, the legitimacy of the opposition of some ANA and ANP to the Taliban is recognised (Hennessey, 2009), as is that of the interpreters on whom ISAF forces rely (Olafson, 2011).

The articulation of ideas about ISAF allies is also constitutive of the geopolitical imaginary which weaves through these books. Whilst other ISAF troops are not explicitly Othered in the way that Afghan allies and enemies are, there remains a sense of their difference. Bury's company of Irish Rangers in Sangin is bolstered in numbers, equipment and expertise by the arrival of a company of US Marines. ‘Their hardware is inspiring. As is their attitude. Like Cupples [an Irish Ranger of US extraction], this is their war. Afghanistan is their Japan; they have a score to settle.’ (Bury, 2010: 138). Other allies, some characterised as ‘non-swimmers’ for contributing little or nothing to the coalition effort (Orchard, 2009), are portrayed in ways that mark out their distinctiveness to British forces and the different rules of engagement and standard operating procedures under which they work. Estonian troops during the attack on Garmsir are found by Beattie ‘doing whatever Estonians do’ during downtime, and writing medical notes in Latin thinking it to be the universal language of medicine (Beattie, 2008). Czech forces celebrate a Born Naked day, which is supported by British troops because it is for charity, but is seen as a little eccentric – Grahame tries ‘not to get an eyeful of any Czech tackle [male genitals]. One look could give you a serious inferiority complex’ (2010: 255), and clearly these Czech warriors' physiques (‘the Czech Army blokes were monsters…’) are a little unsettling. The geopolitical imaginations circulating within these texts are about the preservation of British distinctiveness.

As for Afghan civilians, the residents of Helmand whose security ISAF troops are deployed to ensure, they are largely absent from the memoirs. There is a practical reason for this: the roles undertaken by the people who write the memoirs and the circumstances in which they undertake them preclude regular contact, so although some memoirists at some points have cause to engage with civilians, these encounters are rare. Civilians are a present absence; a number of memoirists mention, almost in passing, the absence of civilian populations either because they have fled settlements permanently (although there is never any discussion as to where they have fled – the reader is just told that they are not there) or because they have lain low during fighting. The present absence of civilians – the missing farmers in fields, missing children the alleys around courtyards, missing customers in bazaars – is reported as an indicator noted at the time of imminent attack. Civilians are also an absent presence, because when they do appear, they are reduced to a flash of a blue burkha, a bright splash of a child's coloured clothing, smudges of browns and greys of a farmer in the distance, or a collective, unfathomable group of old men at village *shuras* discussing local matters with British troops. They are there, but without form or voice. The exception here is Docherty's account; as a Pashtu speaker he at least has a chance of direct communication rather than the mediated and stilted exchanges undertaken through interpreters. They become more visible when wounded; Duncan, towards the end of his account as a Chinook pilot, reveals quite suddenly to the reader that in fact a lot of his time beyond the dramatic incidents recounted in the text is spent flying injured civilians to the local hospital (2011). Beattie opens his second book (2009) with an account of the injury of a civilian child who he is powerless to help. It is primarily through their injuries that civilians become more than ghostly presences.

The people figuring most prominently, of course, are authors' fellow personnel. The memoirs make explicit that for those deployed, the only people who really count are other unit members, and it is these people who essentially provide each other with the only sustainable rationale for fighting (see also Woodward, 2008). As Beattie explains, it was not national identification which drove the Afghans with whom he worked, but rather loyalties at the smaller scale of family, village and tribe, ‘people they knew and respected’. He continues, ‘In that sense they were actually not much different from me. In Afghanistan I wasn't really fighting for Queen and country. I was there for my regiment, for my colleagues, for my friends’ (p. 297), a point echoed by Scott (2008). Overshadowing all these accounts is the loss of colleagues and grief at their absence, and anger at the deaths of young men in a war which makes so little sense to so many of the personnel. This sense of collective experience, and the (im)possibility of articulating this to those who have not shared this experience threads through the memoirs, and we return to the consequences of this below.

### What is this place?

Geopolitics has been described as ‘that ideological process of constructing spatial, political and cultural boundaries to demarcate the domestic space as separate from the threatening other’ (Dalby, 1990: 173). In the memoirs, states of threat and safety are calibrated according to a finely graded, spatialised, affective register in which space is constituted as dangerous or as safely domesticated. As geopolitical representations, they inform a reading of Afghanistan that is more nuanced than the scripts of the country as a space merely of undifferentiated danger implied by much media reporting.

Danger is a question of degree and of geography. The memoirs reflect common modes, points and spaces of entry, movement within and exit from the country, and map these spaces according to levels of threat. Kandahar airbase, the point of entry by air for all troops, is a threshold, a place of transit, a place where threat exists (the base is mortared, for example) and which smells bad because of an open cesspool (‘poo pond’) in the base, but where retrospection sees little that could be described as dangerous. Camp Bastion, the forward location for British troops in Helmand province, a tented city planted in the desert, has an edge of danger, the runway described by Duncan (2011) as ‘a black scar along the belly of a sleeping giant’ (p. 217). The forward operating bases, district centres and platoon houses where units have been based (sometimes besieged) when on operations over the years in the settlements of Helmand – Gereshk, Sangin, Now Zad, Kajaki, Garmsir, Musa Qala, Nad-e-Ali – are the places where the real action occurs. The camps and compounds in which troops live, with their limited utilities and reliance on airborne resupply, heat and cold and dust and mud (depending on the season), Hesco defences and sandbagged sangars provide the backdrop, the stage for unfolding action from which patrols depart and return. They are unsafe in their vulnerability to attack by small arms and mortar fire, and they are made safe only through military engineering, endless shifts of guard duty (‘stag’) and the watchful eyes of Apache helicopter pilots and of other guards in other compounds.

Beyond base walls, the stories of those in infantry roles are tales from the riverbank. The Helmand River which names the province, the feature which makes this place home to the (absent, present) civilian agricultural population, is precisely the source of most of the danger the memoirists encounter. The river flows south, and along its course at depths ranging from metres to several kilometres lies irrigated farmland with crops of maize, wheat and opium poppies, orchards and woodland plantations. Canals, streams and irrigation ditches dissect the land on the banks, crossed by bridges and culverts. Homes enclosed by the hard mud walls of farm compounds connect and cluster, linked by roads, tracks and paths. This is a pre-modern place. Garmsir has

…no steel, glass and brick constructions, architect-designed or with a sense of modernity and function. This was a town built of mud. Single-storey shops and homes with flat roofs, little more than boxes. (Beattie, 2008: 110)

And all these features – the fields, the streams, the dwellings, the infrastructure – are sources of danger. Snipers lurk, ambushes are set, enemy advances are made, improvised explosive devices are laid and soldiers dread to tread. We are imbued as readers of these memoirs with an intimate understanding of the ‘centrality of fear to the last decade's geopolitical order’ (Dittmer & Gray, 2010: 1667).

Amongst the trenches, trees and crops crowding the meandering waterway there was every opportunity for the enemy to lie in wait. The Taliban could be as close as ten metres away in places and we would not have a clue. It frightened the life out of me. (Beattie, 2008: 101)

The memoirists may fear to tread, but tread they must. The memoirs of infantry and other ground-based personnel – Hennessey, Beattie, Docherty, Bury, Scott, Cartwright, Flynn, Farthing, Croucher, Grahame, Olafson, Compton, Ormrod, Taylor – revolve around patrolling. Ground is dominated, the fight taken to the enemy, the patrols initiated to draw fire, buildings cleared only to be given up. Men are killed, injured. Compton (2009) is badly burned when a rocket-propelled grenade ignites his vehicle. Ormrod (2009) loses two legs and an arm when he trips a landmine. Forces are stretched thin, evacuations of the injured are uncertain, landing zones are declared ‘hot’. Fear is spatial, located behind walls and in small depressions on the ground.

Surrounding the green zone, surrounding Bastion, is the desert, the Dasht-e-Margo, the GAFA (the ‘Great Afghan Fuck All’ – Docherty, 2007), the one place where safety can be assured because its very nature as desert defies habitation from all but a few Bedouin herders. From here, safety is assured because advances can be seen – approaching vehicles throw up clouds of dust on the flat barren surface (Scott, 2008). Even here, though, transit is complex; routes to the start-lines for offensive actions must detour to avoid indicating to enemy observers the possible target point. The rocky ground, sand and wadis defy the best attempts of drivers and their vehicles, slowing progress. Such are the dangers of ground-based movement, that as the war progresses new armoured vehicles are introduced and the Chinook helicopters work round the clock on supply and resupply, and the evacuation of the wounded and dead (Duncan, 2011; Hammond, 2009) and the Apache helicopters keep watch (Macy, 2008, 2009; Madison, 2010). These in turn draw fire – both Hammond and Duncan's accounts as Chinook pilots were written around specific actions under enemy fire for which they were both decorated. Contacts pin troops to place; Hennessey (2009) spends 14 h one day, lying on a roof, under fire and returning fire to a group of enemy fighters located in a nearby treeline. Nowhere is safe.

The comparators, reference points drawn to make sense of the conflict, come from other conflicts in other times.

Skirting around poppy fields, we try to avoid falling into the swamps. It still rains, grey and cold. We come to an over-flowing ditch. […] We wade through chest-high water, weapons above our heads, as the rain splashes the water and our faces. *Vietnam*. We cross, slip, laugh and continue on. The search dog and the metal detectors come out, slowly moving, slithering, falling, coating ourselves in the grey-brown slick. *This is warfare at its most basic*. (Bury, 2010: 109, emphases in original)

References are also frequent to Rorke's Drift (in the 1879 Anglo-Zulu war), and the Korean War, identified as the last time British forces engaged in fighting as fierce (Scott, 2008; Tootal, 2009), though as Hennessey notes, ‘presumably to the justified annoyance of those Falkland veterans marking the twenty-fifth anniversary of what was hardly a picnic’ (2009: 15). Interestingly, the 19th century Anglo-Afghan wars are very rarely mentioned and then only in passing, and references to other British counter-insurgency operations are absent. A further reference point is *Star Wars* and a number of memoirs draw the comparison between the terrain of Afghanistan and the fictional terrains shown in the film series. Bury, for example, describes

‘…Do Ab, a desert village of red mud walls and berms built right into the rock face. Pinprick holes of light puncture the curtain of night. It's a scene straight out of *Star Wars*. (Bury, 2010: 75)

The fictional planet home of Luke Skywalker in the first *Star Wars* film is, of course, hot, arid and lawless. For Duncan in his Chinook, one flight becomes ‘a bit *Star Wars*. There seemed to be fire coming from everywhere’ (2011: 236), a re-enactment of the best cinematic efforts of the forces of The Empire.

But there is beauty here. Beattie, taking his turn on guard duty in the desert before the attack on Garmsir, is struck by the peace. ‘Looking up I could see a stellar display from the cosmos. It was spell-binding, mesmerising’ (2008: 85). Farthing (2010) looks to the peaks of the mountains in the distance and fantasises about opening up a climbing and outdoors activity centre there, and of walking the peaks with Nowzad, the dog he has rescued. Duncan, flying Chinook helicopters in and out of Kajaki, marvels at the ‘majesty and breathtaking, forbiddingly beautiful vista of Kajaki from the air’ (2011: 72), and the colours of the lake created from the dammed river, ‘a rich vivid aquamarine that changes from a deep emerald to turquoise, depending on the position of the sun’ (p. 73). Flying over the Helmand River and the Green Zone, he asks himself ‘how can somewhere so pretty be so shit?’ (p. 303).

### Memoirs, geopolitics and militarism

The military memoirs coming out of the Afghanistan conflict are significant as texts through which a geopolitical imaginary about the place and purpose of the war are articulated for wider popular consumption in published form. The ideas we discuss here are not the sole textual features through which this geopolitical imaginary is woven. Less prominent are ideas about the technologies, tools and techniques used by ‘we…futuristic imposters in [these] ancient town[s]’ (Bury, 2010: 98), the effects and affects of violence that mark the bodies and souls of the writers, and the identities and identifications wrought by the experience of war. Underpinning these, and again not explored in depth here, is something of the excitement of war which these books convey and their authors analyse. We have restricted our analysis to the ideas with most prominence which collectively comprise a script about a deployment based on uncertain aims, in an unknowable place, against an elusive and tenacious enemy, conducted through operations in heat and dust which are difficult and dangerous for people who volunteered to do so but who become marked by their efforts, and who are motivated ultimately and only by each other. These books legitimate a specific view of ‘Afghan’ (a term now used widely in popular discourse, emanating from military personnel) as a place and conflict where civilians are absent, opponents are reduced to body counts, and where the complexities of intervention are glossed over in the rush to a hard, fast and brutal military rather than political settlement. We should also note that we have focused in this paper on the commonalities shared across the Afghanistan memoirs as a whole, prioritising features reflective of a shared script constructed and reproduced across the collection of books and originating in a core set of ideas emanating from British military briefings and practices in Helmand. We have not attempted here to tease out the contradictions, ambiguities and differences between the books.

We also note that because of the commercial, institutional and governmental structures through which these books come into being, they prioritise acts categorised as brave, selfless and courageous and are mostly silent about all those other things soldiers do in conflicts which indicate the brutality of the brutalised. There are further, more personal acts of censorship which (as our author interviews indicate) set limits around the degrees of detail authors feel willing and able to provide in the course of exploring the horrors and brutalities of war, particularly when it comes to describing the deaths and injuries of colleagues (and as with any memoir, these are partial accounts). These books are vectors of militarism, mechanisms by which the logic of military intervention and the use of military power comes to be justified and rationalised, and for all the caveats we provide about their partiality, taken as a group offering a metanarrative, we can identify a discourse which normalises the military response to Afghanistan and is celebratory of the individuals deployed to execute lethal violence.

But for all that these books are ‘‘herographies’ and blood-and-guts accounts of derring-do’ (Ledwidge, 2011: 7), they are also about the limits of military intervention and of military power, and about assertions of the humanity of the soldiers tasked with conducting the war. Whilst these accounts make explicit the excitements and thrills of war, they are also about its collective and personal costs, about trauma and survival in the aftermath. They are about the disappointment of being let down by commanding officers and their strategies, about perceptions of absence of domestic civilian support, about equipment shortages. Most starkly, they are about being disappointed by war. For Bury's reader, alert throughout the book that its author is living a childhood dream of soldiering, his sudden realisation during an intense firefight that he just does not want to do this job any longer comes as a jolt. For Hennessey's reader, the breathless excited young subaltern in his first contact is replaced by the emotionally exhausted man leaving Afghanistan haunted by the idea that he is little more than a meddling tourist. So whilst these books are vectors of militarisation, mechanisms by which military ideas, rationales, explanations for action are translated into civilian discourse, they are also vectors of ideas about the soldier and about the lived experience of military participation at odds with the dominant heroic soldier figure circulating in popular discourse (see Woodward et al., 2009) and fed, still, by the genre. There is also something notable about the Afghanistan memoirs in their potential to generate solidarity as an ethical and political response to war, and to this that we now turn.

## Text and *testimonio*

In arguing for the continued salience of the text as a source for understanding geopolitical imaginaries, we want to be clear that we are not dismissing the wider paratextual practices around the text which provide it with its textual and visual threshold (Genette, 1991; Woodward & Jenkings, in press-a) or the value of recent post-textual critiques within the critical geopolitical project highlighted above. Thinking about military memoirs requires thinking about the processes of authoring and publication, marketing and advertising, their materiality and visuality, their consumption by interpretative communities, and their intersections with other popular (and indeed high) cultural forms. But we want to conclude by exploring this question of the military memoir's communicative possibilities, as a way of re-stating the significance of the text in critical geopolitical inquiry and to the wider social world.

The military memoir in its contemporary form sits unproblematically within Western bourgeois literary traditions of coming-of-age stories of the *Bildungsroman* and of picaresque tales which prioritise the singular masculine ‘I’ and his worldview (Harari, 2008). Yet for all that, we conclude that the contemporary memoirs emanating from the Afghanistan war do more than this. Whilst the revelatory and personal is still there (and they are also increasingly about the corporeal and affective experience of war – see Woodward & Jenkings, in press-b), this is increasingly matched and countered by assertions of humanity, collectivity and group solidarity, and these feed into the geopolitical imaginaries and representations of the soldier to which these books contribute. This collectivity is of sufficient novelty within the genre that it requires consideration, and we suggest that there are insights to be brought to our reading of contemporary British military memoirs which can be drawn by looking at these books with reference to *testimonio* literature, ostensibly a very different type of life-writing.

*Testimonio*, a genre of published narratives told in the first person by a protagonist about life experiences in collective struggle, emerged in 1960s Latin America and is associated primarily with movements for national and class liberation (Beverley, 1992, 2004). At first sight, comparisons between *I*, *Rigoberta Menchú* and *Callsign Hades*, or *Si me permiten hablar: Testimonio de Domitila, una mujer de las minas de Bolivia* and *Fire Strike7/9* might seem far-fetched (beyond their respective connections to armed violence), and we are aware that for some readers consideration of the military memoir in parallel with *testimonio* is provocative. But if we consider the military memoir in terms of the defining features of *testimonio*, there are facets to the memoir which become prioritised, allowing us to say something about their wider purchase and communicative power, and in turn something more nuanced about their function as communicative texts about geopolitical imaginaries and the utility (or otherwise) of military force.

In *testimonio* the intentionality and urgency of communication around a problem is paramount in the act of narration. This is a central feature of military memoirs, which revolve around the problem of being caught up in war and dealing simultaneously with its excitements and traumas, and with the sense of absence of civilian understanding of this back home in the UK. Moreover, and unlike the novel, the text of *testimonio* ‘promises by definition to be primarily concerned with sincerity rather than literariness’ (Beverley, 1992: 94). This is also a feature of military memoirs, with their common, stated concerns with accuracy and truth-telling; Hennessey, for example, introduces his narrative with an explicit statement about the honesty of his account. The production process of *testimonio* contributes to its ‘truth-effect’. *Testimonio* frequently involves the recording of oral statements, their transcription, and subsequent editing by an interlocutor (although the processes of production of *testimonio* has fed debates around authenticity). Our research interviews with authors of published memoirs (including six authors of Afghanistan memoirs), show similar production processes involving significant degrees of intervention from co-writers and copy-editors, and the use of recorded oral accounts and documentation, with the resulting text produced collaboratively. Doug Beattie's two books, for example, credit a co-writer Philip Gomm with much of the work involved in shaping Beattie's stories into a form suitable for publication. Table 1 shows the extent of collaborative production as indicated through the differences between title and copyright attribution.

In *testimonio*, there is a pledge of honesty which distances the genre from the picaresque novel. In military memoirs, even whilst the stories are picaresque it is the explanation behind the transgressions, the justification for the rule-breaking, which provides the moral core to their respective stories. In military memoirs, even whilst the stories are picaresque – Farthing was bending regulations by keeping dogs in the Now Zad base, Macy and his Apache crew were defying orders by retrieving the body of Matthew Ford from Jugroom Fort under enemy fire – it is the explanation behind the transgression, the justification for rule-breaking, which provides the moral core to their respective stories. In Farthing's case, he felt unable to help the civilian population directly, but could do so indirectly by trying to control stray dogs. In Macy's case, he felt unable to leave a man to the mercy of potential Taliban capture and violation, hence his efforts to recover Ford (who, at the time of the rescue he thought was still alive). As with *testimonio* the central concern of many of the Afghanistan memoirs is less the troubled or problematic hero or protagonist, and more the problematic collective social situation in which the narrator in placed, specifically a confused and seemingly intractable conflict.

The collective social situation is central in *testimonio* with the narrator speaking for, and in the name of, a community or group. *Testimonio* is by definition democratic and egalitarian, ‘an affirmation of the individual self in a collective mode’ (Beverley, 1992: 95, see also Sommer, 1988). This collectivity is absolutely central to military memoirs. Military activities are collective activities, military groups are bonded groups, the bonding is operationally necessary and deliberately forged during training, but (as the memoirs show time and again) is strengthened, deepened and sustained in operations. Whilst memoirists may frame their books as personal accounts, they are also emphatic about the collective experience around the events described. So just as in *testimonio* where there is an erasure of the function and textual presence of the author (Beverley, 1992), in military memoirs we see the interweaving of others' stories and accounts, reflections on group solidarity, and ultimately a rationale for involvement in armed conflict as service for an identifiable platoon, company or regiment. Scott's *Blood Clot*, for example, is entirely about this collective mode of being; he himself, although telling a story through the first person narrative, continually effaces himself in favour of a narrative foregrounding the experience of his platoon as a whole. Flynn's second book (2011) is based on the interweaving of reports of personal experience with accounts and analysis he records as told directly to him by other serving personnel. Although publication conventions for *testimonio* and military memoir alike emphasise the need for an identifiable single author, in both forms of life-writing, the function of the text is to speak for the experiences of the group. In many memoirs, the narrative thrust is not around the singular experience of the author, but about a collective ‘we’ of a unit and the enactment of community in the face of the larger power structures of the military machine.

In *testimonio*, the erasure of the author and the assertions of non-fictional reality produce a complicity between narrator and reader where the possibility is opened up of solidarity as an appropriate ethical and political response to the stories told. *Testimonio* asks for the readers' identification with a cause and situation otherwise distant and potentially alien to him or her. We see this too in military memoirs, as we have shown with Beattie's appeals for recognition of a cause and Tootal and Docherty's appeals to a civilian public readership to comprehend the almost impossible positions they, as commanding officers, were placed. Our research interviews with authors reinforce this, with authors locating a motivation for writing and publication with a desire to tell their story to a broader public, which is understood by authors to be receptive to the story of the soldiers' lived experience in a context where there is absence of detailed information about the Helmand campaign. In addition, despite our not having data on broad reader responses to memoirs, our close reading and analysis of these books suggests a persuasive capacity to these texts in the extent to which they invite reader identification. Both *testimonio* and the military memoir share an intent around raising consciousness, sharing a lived experience of violence and generating support for a cause and a group. Both *testimonio* and the military memoir argue for recognition of the humanity of the group concerned to a wider public readership. Whilst this capacity within *testimonio* is well-recognised, and often celebrated, this is a less-understood feature of the military memoir, particularly amongst those not overburdened with experience of reading the genre. Yet as Bury puts it, ‘these are your warriors. And this is your war’ (2010: 294), and the appeal is made consistently across these books for public recognition of the soldier and his or her experience.

Above all else, *testimonio* is about the affirmation of the individual experience ‘in connection with a group or class situation marked by marginalization, oppression, and struggle’ (Beverley, 1992: 103; Gelles, 1998; Hanlon & Shankar, 2000). If it loses that, it is not *testimonio*. *Testimonio* ‘always signifies the need for a general social change in which the stability of the reader's world must be brought into question’ (ibid) and provides both representations of and the means by which new forms of subaltern resistance and struggle can proceed (p. 110). *Testimonio* is driven by a desire not be silenced or defeated, and by a ‘desire to impose oneself on an institution of power […] from the position of the excluded and the marginal’ (Beverley, 1992: 96). We should be clear here that it would be stretching the point to make claims for military personnel and their memoirs in exactly these terms; we are not equating the military memoir with *testimonio*. Although frequently marginal and marginalised, personnel serving with the British armed forces experience their social situation in conditions far removed from those endured by the authors of *testimonio*. So whilst many of the memoirists are giving voice to protest from positions of relative subordination within the armed forces, in the face of the power of military command structures to dictate what they will do even when they would chose otherwise, and under the ultimate authority of the political power of the state to initiate armed conflict in the first place, they are afforded some protection to their protest by the legislative contexts in which they operate. Similarly, whilst many memoirists as military personnel are recognised as socially marginalised (and space here precludes a fuller exposition of the marginalisation of the soldier, though see Gee (2007), Edmunds and Forster (2007) and Ashcroft (2012) on this from very different political perspectives), their social protection is ensured by certain rights afforded by the western liberal state and unavailable to most *testimonio* writers.

However, despite falling shy of equating the military memoir with *testimonio*, we would argue that a reading of the memoir informed by an understanding of *testimonio* has validity because of the questions then prompted of the former genre by the latter. To put this another way, whilst *testimonio* does not provide a full model for reading the contemporary military memoirs of Afghanistan, it does show us where to focus in terms of reading the memoir more comprehensively as a source for understanding war. Specifically, we suggest two particular areas for attention.

The first concerns the soldier as geopolitical actor. Put simply, and to echo Dalby (2008), we need to understand the warrior or soldier, this key figure in the material and representational practices around securing and securitizing the West and its ideas of empire. For Dalby, this figure is significant because of his/her role in the violence required to secure the West's identity as the ‘repository of virtue against barbaric threats to civilization’ (p. 440), which in turn prompts questions about the moralities of warfare. How moral codes about violence are played out in extreme circumstances should therefore be a key component of the critical geopolitical project and its attempts to understand – and counter – the reach and realities of empire (Dalby, 2008). As we have illustrated, military memoirs provide a valuable source of data for understanding these moralities. What a reading of military memoirs informed by *testimonio* does is foreground the lived experience, the collective reactions to, and the effects of violence as the critical elements of the memoir texts. Furthermore, such a reading prioritises the negotiations around the moralities of violence which soldiers as simultaneously perpetrators and recipients must necessarily undertake. Put simply, a reading of memoirs informed by knowledge of *testimonio* helps us understand warriors and understand a little more about the things they are required to do in order to understand the links between ‘the conduct of specific warriors and the grand legitimizing narratives of geopolitics that justify putting them in harm's way in the first place’ (Dalby, 2008: 453). The critical geopolitical project needs to attend to the soldier more directly than it has to date in understanding not only the geopolitical imaginaries that they enact through their job, but in understanding also the consequences on them as people.

The second concerns militarism. We suggested at the start of this paper that military memoirs merit critical scholarly attention because of their capacity to both consolidate and endorse, and deny and disrupt, dominant discourses of militarism which affirm the use of organised violence and which prioritise military objectives and rationales. We have stopped short of equating *testimonio* and the military memoir on the grounds that the social and political struggles associated with the former cannot be equated with the social and political struggles explored within the latter. However, a reading of memoirs informed by an understanding of *testimonio* draws our focus on those instances within the memoirs, and the work done by the genre, which speak to the collective struggles of military personnel for visibility and voice and in their expression of loss (Williams, 1993). A salient feature of the Afghanistan memoirs (we see this in memoirs from the Iraq war too) is the communicative urgency of these texts and their authors in laying claim to experiences of loss and trauma as an inevitable but largely unrecognised outcome of military participation (see also McGarry & Walklate, 2011). The Afghanistan memoirs work to recognise, grieve for, memorialise, and mobilise around the idea of loss, of colleagues and of the self. They counter the idea of military solutions as unproblematic, by showing the costs even for those not commonly understood as victims of this war. In short, memoirs have a complex relationship with militarism. If military memoirs are vectors of militarism, they are also vectors of something else.

## Figures and Tables

**Table 1:** Author and publication details for British military memoirs, 2007–2012.

Author citation in text, date	Author attribution, front cover	Author attribution, copyright	Full title	Deployment	Rank, regiment, role	Sales figures, Nielsen Bookscan, February 2011	Publisher
Beattie, 2008	Doug Beattie MC	Doug Beattie and Philip Gomm	An ordinary soldier: Afghanistan: a ferocious enemy. A bloody conflict. One man's impossible mission	Garmsir, 2006, provincial security and co-ordination centre	Captain, 1 Royal Irish, Liaison with ANA, ANP and British forces	48,698	Simon and Schuster
Beattie, 2009	Doug Beattie MC	Doug Beattie and Philip Gomm	Task force Helmand: a soldier's story of life, death and combat on the Afghan front line	Kajaki, Marjah, Attal, 2008	Captain, 1 Royal Irish, OMLT	22,338	Simon and Schuster
Bury, 2010	Patrick Bury	Patrick Bury	Callsign Hades	Sangin, 2008	Second Lieutenant, then Captain, 1 Royal Irish, Infantry role	3095	Simon and Schuster
Cartwright, 2011	James Cartwright	James Cartwright	Sniper in Helmand	Various locations around Helmand, Op Herrick 6	Lance Corporal, sniper trained, 1st Battalion Royal Anglian Regiment	N/A	Pen & Sword
Compton, 2009	Martyn and Michelle Compton with Marnie Summerfield Smith	Martyn Compton, Michelle Compton and Marnie Summerfield Smith	Home from War: how love conquered the horrors of a soldier's Afghan nightmare	Near Musa Qala, recovery back in the UK from severe injuries	Lance Corporal, Household Cavalry, plus female partner	1973	Mainstream Publishing
Croucher, 2009	Matt Croucher GC	Matt Croucher GC with Robert Jobson	Bulletproof: one marine's ferocious account of close combat behind enemy lines	Sangin Valley, 2007	Lance Corporal, Royal Marines	16,965	Century
Dannatt, 2010	General Sir Richard Dannatt	General Sir Richard Dannatt	Leading from the front: the autobiography	Not deployed to Afghanistan, but responsible for overall deployment of British forces	Chief of the General Staff, 2006–2007, London-based	22,053	Bantam
Docherty, 2007	Leo Docherty	Leo Docherty	Desert of death: a soldier's journey from Iraq to Afghanistan	Sangin, 2006	Captain, Scots Guards	8624	Faber & Faber
Duncan, 2011	Flt Lt Alex ‘Frenchie’ Duncan DFC	Flt Lt Alex ‘Frenchie’ Duncan DFC with Antony Loveless, copyright Antony Loveless	Sweating the metal: flying under fire, a Chinook pilot's blistering account of life, death and dust in Afghanistan	Bastion and Khandahar, various missions in Helmand	Flight Lieutenant, Chinook Pilot, Royal Air Force	N/A	Hodder & Stoughton
Farthing, 2010	Pen Farthing	Pen Farthing	One dog at a time: saving the strays of Afghanistan	Now Zad, 2006–2007	Sergeant, Royal Marines	47,185	Thomas Dunne Books, St Martin's Press
Flynn, 2010	Mick Flynn	Mick Flynn CGC, MC, with Will Pearson. Copyright La Joya Ltd.	Bullet magnet: the true story of the most highly decorated serving soldier in the British army	Helmand Province, various, and Musa Qal'ah	Squadron Corporal Major, Blues and Royals, Household Cavalry Regiment	10,498	Weidenfeld & Nicolson
Flynn, 2011	Mick Flynn	Mick Flynn CGC, MC, with Will Pearson. Copyright La Joya Ltd.	Trigger time	Sangin, Musa Qal'ah, Now Zad, Marjah, 2006, 2008	Squadron Corporal Major, Blues and Royals, Household Cavalry	N/A	Orion Books
Grahame, 2010	Sgt Paul ‘Bommer’ Grahame and Damien Lewis	Damien Lewis and Paul Grahame	Firestrike 7/9	Various, unspecified locations, Helmand	Joint Terminal Attack Controller (JTAC), Sergeant, Light Dragoons, embedded with 2 Mercian Regiment, Infantry	12,221	Ebury Press
Hammond, 2009	Major Mark Hammond, DFC Royal Marines	Major Mark Hammond DFC RM with Clare Macnaughton. Copyright Clare Macnaughton	Immediate response	Helmand, Bastion and Khandahar based	Major, Royal Marines, Chinook Pilot with Fleet Air Arm	14,975	Michael Joseph
Hennessey, 2009	Patrick Hennessey	Patrick Hennessey	The junior officers' reading club: killing time and fighting wars		Second Lieutenant, The Grenadier Guards	73,343	Allen Lane
Macy, 2008	Ed Macy	Ed Macy	Apache	Helmand, Bastion and Khandahar based	Apache Pilot and Weapons Officer; WO1, 656 Squadron, Army Air Corps	96,369	Harper Press
Macy, 2009	Ed Macy	Ed Macy	Hellfire	Helmand, Bastion and Khandahar based	Apache Pilot and Weapons Officer, WO1, 656 Squadron Army Air Corps	20,945	Harper Press
Madison, 2010	Charlotte Madison	Charlotte Madison	Dressed to kill: the true story of a woman flying under fire	Helmand, Bastion based	Apache Pilot, Captain 656 Squadron, Army Air Corps	8232	Headline Review
Olafson, 2011	Jake Olafson	Jake Olafson	Wearing the green beret: a Canadian with the royal marine commandos	Various locations around Helmand, 2007	Royal Marine Commando	N/A	McClelland & Stewart
Orchard, 2009	Commander Ade Orchard RN with James Barrington	Copyright Adrian Orchard and James Barrington	Joint force harrier	Helmand province; based Kandahar Airfield, 2006	Commander, 800 Naval Squadron, Fleet Air Arm, flying Harrier GR7	35,740	Penguin
Ormrod, 2009	Mark Ormrod Royal Marine	Mark Ormrod Royal Marine	Man down	FOB Robinson on injury	Royal Marine Commando	7613	Bantam Press
Scott, 2008	Jake Scott	Jake Scott	Blood clot: in combat with the patrols platoon, 3PARA, Afghanistan 2006	Various locations around Helmand, 2006	Corporal, 3rd Battalion The Parachute Regiment	1681	Helion
Taylor, 2011	Chantelle Taylor	Chantelle Taylor	Bad company: a woman face to face with the Taliban	Helmand, 2006, Nad-e-Ali, 2008	Sergeant, Combat Medic, attached to Infantry regiment	N/A	DRA Publishing
Tootal, 2009	Colonel Stuart Tootal DSO OBE	Colonel Stuart Tootal	Danger close: commanding 3PARA in Afghanistan	Various locations around Helmand, 2006	Full Colonel, Commanding Officer, 3rd Battalion, the Parachute Regiment Battlegroup	19,511	John Murray
